# Children's Health Status: Examining the Associations among Income Poverty, Material Hardship, and Parental Factors

**DOI:** 10.1371/journal.pone.0000940

**Published:** 2007-09-26

**Authors:** Godwin S. Ashiabi, Keri K. O'Neal

**Affiliations:** Department of Human Development, California State University East Bay, Hayward, California, United States of America; Harvard School of Public Health, United States of America

## Abstract

**Background:**

We examined a model of multiple mediating pathways of income poverty, material hardship, parenting factors, and child health status to understand how material hardship and parental factors mediate the effects of poverty on child health. We hypothesized that: (a) poverty will be directly associated with material hardship, parental depression, and health status, and indirectly with parenting behaviors through its effects on parental depression and material hardship; (b) material hardship will be associated with parental depression, parenting behaviors, and health status; and (c) parental depression will be correlated with parenting behaviors, and that both parental depression and parenting behaviors will predict child health.

**Methods and Results:**

We used data from the 2002 National Survey of American Families for a sample of 9,645 6-to-11 year-olds to examine a 4-step structural equation model. The baseline model included covariates and income poverty. In the hardship model, food insufficiency and medical need were added to the baseline model. The parental model included parental depression and parenting behavior and baseline model. In the full model, all the constructs were included. First, income poverty had a direct effect on health status, and an indirect effect through its association with material hardship, parental depressive affect, and parenting behaviors. Medical need and food insufficiency had negative effects on child health, and indirect effects on health through their association with parental depression and parenting behaviors. Finally, parental depression and parenting behaviors were associated with child health, and part of the effect of parental depression on health was explained by its association with parenting behaviors.

**Conclusions:**

Poverty has an independent effect on health, however, its effects are partially explained by material hardship, parental depression and parental behaviors. To improve children's health would require a multi-pronged approach involving income transfers, health insurance coverage, food and nutrition assistance, and parenting interventions.

## Introduction

Children's health is influenced by a confluence of factors, such as family income, health care, biological, behavioral, and sociocultural [1]. However, the mechanisms through which these factors interact to affect child health are still poorly understood. To aid in examining the processes involved in child health we draw on three aspects of earlier research; in particular, research on income-poverty, material hardship, and parental psychological resources (parental depressive affect and parenting behaviors) and their associations with child health. Our goal in this investigation is to extend past analyses by integrating constructs from these three aspects to understand how they interact to effect child health. We begin our review by examining research linking income poverty to child health, material hardship, and parental psychological resources. Pursuant to that, we examine the implications of research on material hardship for child health and parental well-being. Finally, we discuss findings on the links between parental well-being and child health, and propose a causal model of the associations among income poverty, material hardship, parental psychological resources, and child health.

Our argument is that income poverty has a direct effect on material hardship, parental depressive affect, and child health, but an indirect effect on parenting behaviors. Furthermore, we argue that material hardship, parental depressive affect and parenting behaviors will mediate the effect of income poverty on child health status. Our intent is to understand how material hardships and parental psychological resources mediate the effect of income poverty on child health. This endeavor will deepen our understanding of the mechanisms involved in the production of child health, and be useful for policy as it relates to federal income transfer or in-kind programs and practice specific to parent intervention efforts.

### The Income Poverty Perspective

Past research suggests that income poverty is associated with child health [1], [2], [3], material hardship [4], [5], [6], and parental depressive affect [7], [8], [9]. Poor children more commonly experience respiratory infections [10], gastrointestinal problems [11], general ill health and nutritional deficiencies [12], accidental injuries [13], suffer disproportionately from almost every disease, and show higher rates of mortality than do their nonpoor counterparts [13], [14]. Conversely, higher family income enables parental investment in health promotion [15] that lead to better child health [16].

Poverty has also been linked to material hardships, such as, food insecurity/insufficiency and medical need [4], [5], [6]. While Short [17] found a relatively low association between income poverty and lack of medical care, Iceland and Bauman [6] reported that income poverty was more strongly associated with food insecurity, difficulty paying bills, and possession of consumer durables, than with housing and neighborhood problems, and fear of crime. And Newacheck, Stoddard, Hughes and Pearl [18] found that medical needs, such as lack of health insurance, affected health care usage; uninsured children did not have a regular source of care and were more likely to have go without needed medical, health, or dental care.

Numerous studies have shown that income-poverty is associated with parental depressive affect, and indirectly with parenting behaviors [19], [20], [21], [22]. Conger et al. [7] reported that parental depression mediated the effect of per capita income and unstable work on adolescents' adjustment through its effects on disrupted parenting behaviors. Similarly, Yeung et al. [22] found that maternal emotional distress and parenting behaviors mediated the effects of income on children's behavior problems; while McLoyd et al. [19] reported that economic stressors adversely affected adolescent socioemotional well-being indirectly through their impact on mothers' psychological functioning, parenting behaviors, and mother-child relations.

Using income as an alternative for total family resources may misrepresent the resources that are actually available to a family for meeting its basic needs. This is because families' living conditions are determined by more than current income, and families may experience standards of living for reasons not explained by current income [23], [24]. Thus, to the extent that families are able to meet their basic needs using accumulated wealth, credit, or other sources, measures based on income will likely misrepresent families' situations [4], [23], [25]. In view of this limitation, several researchers [4], [17], [25] have argued that it is important to assess not only the effects of income, but also of any material hardship that (may) accompany income poverty [4], [17], [25]. Proponents of material hardship measures view them as an important complement to income-based measures that provide a different portrait of the extent to which families are able to meet their basic needs [23].

### Material Hardship Explanation

The second area of research suggest that material hardship [4], [6], [17], [23], such as, food insecurity/insufficiency [26], [27], [28], [29], [30], [31] and medical need [32], [33], [34], [35], [36] have implications for parental depressive affect, parenting behaviors, and children's health. Alaimo et al. [26] found that independent of other factors, food-insufficient children were significantly more likely to have poorer health status and to experience more frequent stomachaches and headaches than food-sufficient children; preschool food-insufficient children had more frequent colds. Further, food insecure children have been found to have odds of fair/poor health nearly twice as great, and odds of being hospitalized a third as great compared with food-secure children [29].

Mothers who report food insecurity are more likely to have a diagnosis of posttraumatic stress disorder [31], higher odds of experiencing major depression and distress [37], [38], generalized anxiety disorder [39] and increased risk of depression [40]. In a qualitative study, Hamelin, Habicht, and Beaudry [41] found that food insecurity results in disrupted household dynamics evidenced by parental irritability, anger, parental unavailability, and conversation gap with children. Similarly, Ashiabi and O'Neal [42] found that higher levels of food insecurity predicted heightened parental depression, and a reduction in positive parenting behaviors.

Studies that have focused on medical need show that insurance status predicts children's health-care usage [32], [33], [34], [35], [36]. Uninsured children compared with their insured peers have fewer physician visits [33], [35], [36]; are more likely to go without any physician contact in a given year [33], [34]; to receive inadequate preventive care [32], to be without a usual source of care [33]; and less likely be seen by a physician when they have symptoms of illnesses that warrant physician office visits [43]. Paul et al. [35] studying the effects of health insurance on children's access to primary care, concluded that having health insurance is strongly associated with access to care. Other research has shown that poor children have higher rates of hospitalization for illness or injuries [44] which are generally indicative of inadequate primary care [45].

The mechanisms through which food insecurity/insufficiency and other forms of hardship affect children's health are not clear. Food insufficiency may affect child health through such means as reduced food intake, food quality, and micronutrient deficiencies [26], [29]. If the nutritional quality and frequency of meals in food-insufficient households were reduced to such an extent that micronutrient deficiencies result, or if the variety of foods available in food-insufficient households were severely constrained resulting in malnutrition [29], any one of these conditions or a combination of them could explain the link between food insufficiency and health status. Moreover, medical need, such as, postponing medical care or not purchasing medication because of financial constraints has a direct bearing on children's health status. It is also plausible that the stress associated with medical need or food insufficiency may increase parental stress and depressive symptoms that adversely affect the quality of parenting behaviors that are relevant to child health. Previous research has not explicitly tested the relationship between medical need and parental depressive affect and parenting behaviors.

Another limitation is that studies that have used material hardship measures have generally focused on only one type of hardship; however, examining multiple dimensions of hardship allows us to understand how each form may be associated with income poverty and child health. Other studies [8], [20] have used a latent variable approach and loaded disparate indicators of hardship onto a single latent construct labeled “material hardship.” Such an approach makes it hard to determine which aspect of hardship is associated with child outcome. We extend previous analyses by disaggregating two dimensions of material hardship (food insufficiency and medical need) that have been found to be associated with child health. By doing this, it makes our findings meaningful and interpretable when aspects of material hardship are discussed, and thus expands the discussion on material hardship and our understanding of how varied forms of hardships may be associated with child health.

### Parental Psychological Resource Explanation

Finally, research in the parental psychological resource domain suggests that parental depression and parenting behaviors have implications for child health [46], [47], [48]. Specifically, depressed parents view themselves as inadequate parents [46], as having little control over their children's development [49], and are critical of their children and perceive them in a negative light [5]. Depressed parents also provide less quantity and poor quality care to their children, and are less responsive to them [50], [51], [52]. Furthermore, parental depression negatively effects disease management and help-seeking behavior for health problems of children [47], [53]; children of depressed parents are less likely than children of non-depressed parents to obtain the needed health care [48], [54], and to suffer from acute illness symptoms [53]. In essence, parental depression effects quality of parenting and parental behaviors that compromise the health of children [52], such as, not seeking care on time and not administering medications to a child [55], [56].

The parental psychological line of research does not explicitly examine and explain how income poverty and material hardship may be associated with parental depression affect and parenting behaviors. Drawing on the family economic stress framework [7], [9] which suggests that income poverty affect children by limiting their access to material resources, and indirectly by increasing parental depressive symptoms and poor quality parenting, we argue that income poverty and material hardship will be associated with parental depression and parenting behaviors. We contend that income poverty and material hardship may elevate levels of parental depression [8], [31], [37], [40] and adversely affect quality of parenting behaviors [8], [41], [42], and ultimately child health [52].

### Research hypotheses

**Figure 1:**
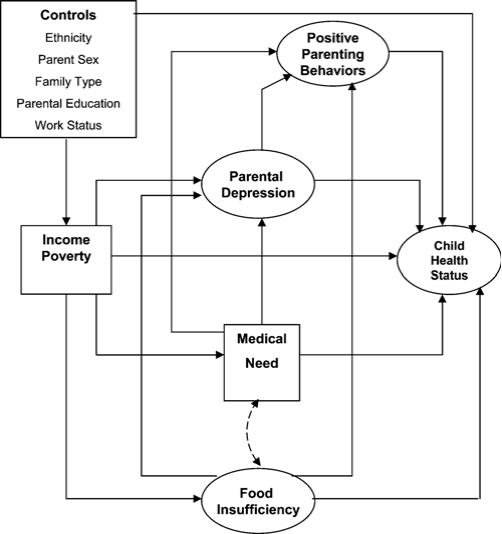
Model linking Income, Material Hardship, and Parenting to Child Health Status.

Given the literature reviewed we propose a causal model (Figure 1) to examine the multiple mediating pathways of income-poverty, material hardship (food insufficiency and medical need), parental depression, positive parenting behaviors, and child health status. A structural equation model is suitable for this investigation because it allows the simultaneous examination of the causal relationships among latent variables, observed/manifest variables or both, and enables an examination of indirect effects. We hypothesize that: (a) income poverty will be directly associated with material hardship, parental depression, and health status, and indirectly with parenting behaviors through its effects on parental depression and material hardship; (b) material hardship will be associated with parental depression, parenting behaviors, and health status; and (c) parental depression will have a correlation with parenting behaviors, and that both parental depression and parenting behaviors will predict child health.

## Methods

### The Data and Sample

We used secondary data from the 2002 National Survey of American Families (NSAF) data set (a survey of the economic, health, and social characteristics of children and adults under the age of 65, and their families). Given that our study involved secondary analysis of survey data, institutional ethics review was not required. The sample for the NSAF is representative of the civilian noninstitutionalized population under age 65 in 13 states and the rest of the nation. The 13 states account for over half of the U.S. population and represent a broad array of government programs, fiscal capacity, and demographic characteristics [57]. The sample consisted of a random-digit dial survey of households with telephones (main frame), and a supplemental area probability sample of households without telephones, and an oversample of low-income households with children. All households screened as low-income and as having children were administered an extended interview. Higher-income households with children and all household without children but with a resident under the age of 65 were subsampled at varying rates prior to in-depth questioning.

#### Data collection

Data collection was conducted from February 2002 through October 2002. Interviewing was conducted in two stages: first, a five-minute screening interview was conducted to determine household eligibility for extended interview, followed by a 27- to 50-minute extended interview. Households selected for an extended interview were sent a brochure describing the study, and received a telephone call at which time they gave their oral consent to participate in the study. If initial contact with a household was not successful, that household was sent another letter reminding them to expect a phone call, and a contact number to call if they preferred to set up an appointment. Telephone interviewers conducted all interviews using computer-assisted telephone interviewing. For households without telephones, in-person interviewers provided cellular telephones to connect them with interviewing centers [57]. In households with children under the age of 18, up to two children were sampled for in-depth study; one under the age of 6 and another between the age of 6 and 17. Interviews were conducted with the most knowledgeable adult—in a majority of cases this adult was a mother, and hereafter referred to as the parent. For this study, data on 9,645 6-to-11 year-olds were used. Children who were reported to have pre-existing developmental health problems were excluded from the final sample to prevent confounding. Descriptive statistics of the sample and measures are reported in Table 1.

**Table 1:** Unweighted Descriptive Statistics of Study Sample.

	Mean (SD)	N	Range
**Family Structure**			
# Lives with no parent		309	1–4
# Lives with single parent		2566	1–4
# Lives in blended (step) family		785	1–4
# Lives in biological/adoptive family		5985	1–4
**Race/Ethnicity**			
# African Americans		1357	1–3
# Hispanics		1621	1–3
# Whites		6667	1–3
**Demographic Variables**			
# Boys		4702	1–2
# Girls		4943	1–2
# Mothers		7904	1–2
*#* Fathers		1741	1–2
Child's age	8.39 (1.72)	9645	6–11
Parental age	37.19 (7.61)	9645	16–85
Parental work status	159 (.75)	9645	1–3
Parental education level	7.23 (2.96)	9645	1–12
Income poverty	2.87 (1.19)	9645	.50–4.00
**Material Hardships Constructs**			
Food Insufficiency Items			
Worried food would ran out	.25 (.42)	9645	0–1
Food bought did not last	.19 (.38)	9645	0–1
Frequency of cutting/skipping meals	.12 (.32)	9645	0–1
Medical need	.06 (.32)	9645	0–3
**Parental Depression Items**			
Very nervous in last month	1.76 (.95)	9645	1–4
Felt calm and peaceful in last month	2.41 (.99)	9645	1–4
Felt downhearted in last month	1.69 (.86)	9645	1–4
Was a happy person in last month	2.18 (.88)	9645	1–4
Could not be cheered up in last month	1.26 (.57)	9645	1–4
**Positive Parental Behaviors Items**			
Child much harder to care for than most	3.70 (.58)	9645	1–4
Child really bothers parent a lot	3.45 (.60)	9645	1–4
Parent gives up more for child's needs	3.45 (.86)	9645	1–4
Parent feels angry with child	3.35 (.86)	9645	1–4
**Child Health Status**			
Past health compared with current health	3.27 (.67)	9645	1–5
Current health status	4.39 (.82)	9645	1–5
Child has injuries	187 (.34)	9645	1–2

### Measures

#### Covariates

Parental education was categorized in 12 levels from completed 8th grade [1] to graduate/professional degree [12]. The mean years of schooling completed was about 7 (that is, up to the vocational or technical certificate level). Race/ethnicity, coded African American (1), Hispanic (2), white (3). Family structure was coded living with no parent (1), lives with a single-parent (2), lives in a blended/step family (3), and lives in a biological family (4). Parents' sex was coded female (1) and male (2). Parental work status was available in the NSAF data and coded not working (1), looking for work (2), and working (3).

#### Income poverty

This was a constructed variable available in the NSAF. It compared family income received in 2001 to the Census Bureau's Federal poverty thresholds for the year, given household composition and family size. To determine income, questions were asked about the amount of money income received by each person in the family, 15 years old and over. Sources of income included, for example, money wages or salary, net income from self-employment, social security, and supplemental security income. This income poverty was categorized in six levels from less than 50% of 2001 poverty line (.5) to equal to or greater than 300% of 2001 poverty line [4]. The mean for income poverty was about 3 (200⇐income-to-poverty ratio⇐300).

#### Material hardship measures

Two constructs were used to assess material hardship: food insufficiency and medical need. This decision was based on the fact that there is no consensus on what constitutes material deprivation [4], [23]. It was also an attempt to assess aspects of hardship with items that had face validity; were the result of financial constraints, not individual taste or preferences;^4^ and would be related to the outcome [23]. Four questions in the NSAF, two of which were combined into two variables (resulting in three variables) were used to indicate food insufficiency as a latent construct. A preliminary question which asked respondents whether in the previous 12 months they had cut or skipped meals (*yes* = 1 or *no* = 2) was combined with a follow-up question that asked how often they had cut/skip meals for lack of money if they answered *yes* to the preliminary question. The follow-up question was answered almost every month (1), through only 1 or 2 months (3). The questions were combined and reverse coded (0 = no through 3 = almost every month) so that higher scores reflected increased frequency of cutting/skipping meals. The scale was then dichotomized (0 = no; 1 = 1 or 2 months to almost every month) to be consistent with the way the USDA scores the food security measure. The third question asked whether food bought did not last. It was answered often true (1) to never true (3). This item was also reverse coded (never true = 1 to often true = 3) so that a higher score reflected increased food insecurity, and dichotomized (0 = never; 1 = sometimes or often true). The fourth question asked respondents if they worried whether food would run out. This was answered often true (1) to never true (3). This item was reverse coded (never true = 1 to often true = 3) so that higher scores indicated increased food insecurity, and dichotomized (0 = never; 1 = sometimes or often true).

Three items in the NSAF were combined to create an index of medical need per suggestions of Beverly^4^ and Oullette et al [23]. The items asked whether in the past year respondents had postponed medical care (yes = 1, no = 2), dental care (yes = 1, no = 2), or medication purchases (yes = 1, no = 2). Prior to scale creation, the items were recoded (no = 0, yes = 1), with scale scores ranging from not experienced any of the medical need (0) through experienced all three medical need (3). Each item had a weight of one. A higher score indicates experiencing more medical need.

#### Parental depression

This latent variable does not measure depression in a clinical sense, but rather parental depressive affect. The five items used asked the parent how often in the past month the parent (a) had been a very nervous person, (b) felt calm or peaceful, (c) felt downhearted and blue, (d) had been a happy person, and (e) felt so down in the dumps that nothing could cheer him/her up. The response categories were all of the time (1) to none of the time (4). Responses to questions about been a nervous person, felt downhearted and blue, and felt so down in the dumps that nothing could cheer him/her up were reverse coded. A higher score on this construct indicates higher levels of depression.

#### Positive parenting behaviors

This latent construct was indicated by four items that asked the parent how often in the past month the parent felt (a) the child was much harder to care for than most, (b) the child did things that really bothered the parent a lot, (c) he or she was giving up more of his/her life to meet the child's needs than he/she ever expected, and (d) angry with the child. The response categories were all of the time (1) to none of the time (4). A higher score indicates positive parenting behaviors.

#### Child health status

This latent construct was indicated by three items, two of which were subjective parental reports of global health status. Subjective measures have been used in maternal ratings of children [58]. and they offer a way of assessing perceptions of health by combining the subjective experience of acute and chronic diseases and feelings of well-being [59]. The first item compared current status to past health on a 5-point scale (much better = 1 to much worse = 5). The second item rated current health (excellent = 1 to poor = 5). Both items were reverse-coded, so that a higher score indicates better health status. The objective measure of health asked parents if a child has had any accidents or injuries in the past year (yes = 1 and no = 2).

### Data Imputation and Analytic Procedure

Various simulation studies have shown that imputing missing data using maximum likelihood (ML) estimation produces better estimates than does listwise or pairwise deletion, or mean imputation [60], [61]. Thus, the expectation-maximization (EM) function in SPSS Missing Values Analysis (which computes ML estimates) was used for missing data imputation. The exact procedures followed are described by Hill [62]. After data imputation, the model (Figure 1) was tested using a 4-step structural equation model (SEM) strategy based on maximum likelihood estimation with the AMOS 6.0 program [63]. SEM model testing examines the degree to which a hypothesized model agrees with the observed data, and also facilitates the simultaneous consideration of associations among latent constructs and observed variables in a model as well as indirect effects, while taking into account covariates.

In Step 1, the baseline model (covariates and income poverty) was used to examine the direct effect of income poverty on child health. In step 2 (hardship model), the material hardship constructs were added to the baseline model. This was used to examine the change in the effects of income poverty and material hardship measures on child health. Also, they were used to look at the effects of income on the material hardship. In Step 3 (parental model) parental depression and parenting behaviors were added to the baseline model (minus the hardship measures). As before, this model was used to examine the effects of income poverty and parenting constructs on health status. Additionally, the model was used to examine the association of income poverty with parental depression, and the correlation between parental depression and positive parenting behaviors. Finally, in Step 4 (full model), the direct and indirect associations among all the constructs in the model were examined.

#### Test for mediation

A three-step procedure for identifying mediation in SEM was followed [64]. Given a predictor variable (X), a dependent variable (Y), and a mediator (M), the first step is to examine the direct X→Y model for adequate fit. The second step is to test the X→M→Y model for adequacy of fit; assuming adequate fit, the model is X→Y, X→M, and M→Y are examined for significance. The final step is to assess the fit of the mediational model (X→M→Y) when (a) the X→Y path is constrained to be zero, and (b) the X→Y is unconstrained. If the unconstrained model does not fit better than the constrained model, the X→Y is reduced to nonsignificance, then full mediation is concluded [64].

#### Goodness-of-fit

The fit indices were used to determine whether the model being tested should be accepted or rejected. Fit indices rule out bad models but do not prove good models. The χ^2^ statistic was used an omnibus test. However, given that the χ^2^ value is affected by sample size, the Comparative Fit Index (CFI) and the Root Mean Square Error of Approximation (RMSEA) for which a test for close fit (PCLOSE) is calculated were used to augment the χ^2^ value. For a good fit, Hu and Bentler [65] suggested a CFI value of at least .95, and RMSEA of *p*<.06; and Loehlin [66] argued for PCLOSE of *p*>.05.

## Results

### Summary of 4-Step Modeling

**Figure 2:**
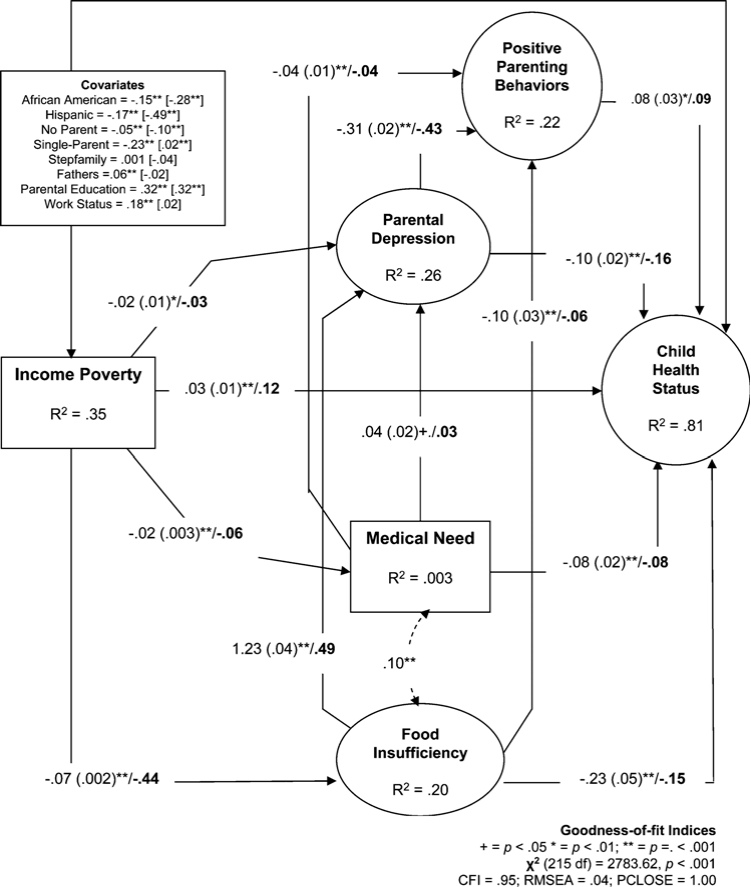
Model of Income Poverty, Material Hardship, Parenting, and Health Status. Unstandardized coefficients (SE), and standardized coefficients (bold face) are reported. Note. Covariate coefficients are reported for poverty and [health status].

The results of the 4-step SEM are reported (Table 2), after which findings for the full model are presented (Figure 2). As shown (Table 2), the baseline model had a good fit to the data using conventional criteria for goodness-of-fit. This finding suggests when we adjust for various factors, income poverty has an independent effect on child health status. The baseline model accounted for 78 percent of the variation in the outcome, and showed that higher income was associated with better child health status (β = .20). When material hardship measures were added to the baseline model (Step 2), the model showed an adequate fit to the data. Also the strength of effect of income poverty on child health status was reduced (β = .12); a .08 decrease in strength of association (about a 40 percentage-point decrease). This model which accounted for 80 percent of the variation in health status also showed that higher levels of food insufficiency (β = −.24) and medical need (β = −.08) were associated with poor health status.

**Table 2:** Summary of 4-Step Structural Equation Model Building.

Steps in the Model	Child Health Status
	Goodness-of-fit
**Step 1: Baseline Model (Covariates+Family Poverty)**	
Income Poverty β (SE)^a^	.20 (.01)**
R^2^ for the outcome	.78
**Goodness of fit indices: χ^2^** (df) for model	87.18 (18 df), *p*<.001
CFI/RMSEA/PCLOSE	.99; .02; 1.00
**Step 2: Hardship Model (Baseline+Hardship Measures)**	
Income Poverty β (SE)^a^	.12 (.01)**
Food Insufficiency β (SE)^a^	−.24 (.04)**
Medical Need β (SE)^a^	−.08 (.02)**
R^2^ for the outcome	.80
**Goodness of fit indices: χ^2^** (df) for model	965.78 (64 df), *p*<.001
CFI/RMSEA/PCLOSE	.96; .04; 1.00
Summary of χ^2^ difference test for mediation	**χ^2^** (1df) = 19.15, *p*<.001
**Step 3: Parental Model (Baseline+Parental Depression+Positive Parenting Behaviors)**	
Income Poverty β (SE)^a^	.16 (.01) **
Parental Depression β (SE)^a^	−.20 (.02)**
Positive Parenting Behaviors β (SE)^a^	.10 (.03)**
R^2^ for the outcome	.81
**Goodness of fit indices: χ^2^** (df) for model	2017.35 (137 df), *p*<.001
CFI/RMSEA/PCLOSE	.95; .04; 1.00
Summary of χ^2^ difference test for mediation	**χ^2^** (1df) = 38.58, *p*<.001
**Step 4: Full Model (Baseline+Hardship Measures+Parental Factors)**	
Income Poverty β (SE)^a^	.12 (.01)**
Food Insufficiency β (SE)^a^	−.16 (.05)**
Medical Need β (SE)^a^	−.08 (.02)**
Parental Depression β (SE)^a^	−.04 (.02)**.
Positive Parenting Behaviors β (SE)^a^	.09 (.03)**
R^2^ for the outcome	.81
**Goodness of fit indices: χ^2^** (df) for model	2783.62 (215 df), *p*<.001
CFI/RMSEA/PCLOSE	.95; .04; 1.00
Summary of χ^2^ difference test for mediation	**χ^2^** (1df) = 19.00, *p*<.001

* *p*<.01; ** = *p*<.001; ^a^ = Standardized β; SE = (standard error).

In Step 3 (parental model), parental depression and positive parenting behaviors were added to the baseline model (minus the hardship measures). This model had a good fit to the data by conventional goodness of fit criteria. As before, the effect of income poverty on health status was reduced (β = .16); a .04 decrease in strength of association, but the decline was not as large compared with that for the hardship model (about a 20 percentage-point decline). The results also showed that increased parental depression was associated with poor health status (β = −.20), whereas positive parenting behaviors were predictive of better health status (β = .10). This model accounted for 81 percent of the variation in the outcome.

#### The full model

The goodness-of-fit for the full model (Step 4), showed that it had a good fit to the data [**χ^2^** (215 df) = 2783.62, *p*<.001; CFI = .95; RMSEA = .04; PCLOSE = 1.00]. This implies that the hypothesized casual model of the associations among the constructs is tenable. The full model explained 81 percent of the variation in health status. In terms of the covariates (Figure 2), being an African American (β = −.15) or Hispanic (β = −.17); living with no parent (β = −.05), or in a single-parent family (β = −.23) were all associated with lower income. On the other hand, being a father (β = .06), having more years of education (β = .33), and having employment were predictive of higher income (β = .06). Furthermore, African American children (β = −.28), Hispanic children (β = −.49), and those living with no parent (β = −.10) were all likely be experience poor health. However, parental education (β = .32) was associated with better health status

For the full model, higher income was associated with better health status (β = .12), and lower levels of food insufficiency (β = −.44), medical need (β = −.06), and parental depression (β = −.03). An inspection of the path weights revealed that income poverty had differential associations with the indicators of material hardship; specifically, higher income had a much stronger effect on reducing food insufficiency than in reducing medical need. In terms of the association between material hardship and parental factors and health status, the results showed that higher levels of food insufficiency (β = −.15) and medical need (β = −.08) were predictive of poor health status. Also, heightened food insufficiency (β = −.06) and medical need (β = −.04) were associated with diminished positive parenting behaviors, and with increased parental depression (β = .49 and β = .03), respectively. Taken together, these findings suggest that both food insufficiency and medical need have adverse effects on child health status and positive parenting behaviors, and increase the risk of parental depression. Finally, heightened parental depression was associated with diminished positive parenting behaviors (β = −.43), and with poor health status (β = −.16), whereas positive parenting behaviors (β = .09) were associated with better health status. These findings, taken together suggest that parental depressive affect has an independent effect on health, but that some of its effects pass through its association with parenting behaviors.

#### Mediation and comparative effects

The test of mediation revealed that material hardship and parental factors partially mediated the effects of income poverty on health status using chi-square difference tests (Table 2). The results suggest that income poverty had an indirect effect on health status (β = .12) as did food insufficiency (β = −.10), medical need (β = −.01), and parental depression (β = −.04), with the effects of income poverty and food insufficiency being the strongest. In order to place the relative magnitude of income poverty in the context of material hardship and parenting factors, the standardized total association between each construct and health status was examined. The findings showed that food insufficiency (β = −.25), income poverty (β = .24), and parental depression (β = −.20) had the strongest total effects on health status than parenting behaviors (β = .08), medical need (β = −.09).

## Discussion

The objective of this study was to examine a model of multiple mediating pathways of income poverty, material hardship (food insufficiency and medical need), parenting factors (parental depression and positive parenting behaviors), and child health status. The findings revealed that material hardship constructs and parenting factors partially mediated the effects of income poverty on child health status. Specifically, without material hardship measures and parenting factors in the model, an increase in family income was associated with a larger effect on health status; however, when material hardship measures were included in the model, the effect of income poverty on health status was reduced by about 40 percentage-points (from β = .20 to β = .12). These findings are consistent with the literature that shows that although income may exert a direct effect on children's well-being, its effects are dampened when mediator variables are included in a model [8], [67]. Taken together, these results suggest that although income poverty has a direct association with health status, its other effects operate through its association with material hardship and parental factors.

Higher family income was also associated with a reduction in levels of parental depression. This is consistent with research that has shown that income poverty influences parental depressive affect [7], [8], [19]. Furthermore, as income increased it had a much stronger effect on reducing food insufficiency than it did medical need. That income poverty had differential effects on the two measures of material hardship is consistent with research finding that income poverty may have differential effects on various dimensions of material hardship [6]. This suggests that material hardship is a multidimensional construct [4], [6], [23]. Thereby, arguing against the practice of loading different dimensions of material hardship (e.g., inadequacy of medical care, housing problems, and food insecurity/insufficiency) onto one latent construct labeled material hardship [8] because it prevents us from understanding how each form of hardship is different from, and differentially effects parents and children.

Increases in food insufficiency and medical need were associated with heightened parental depression and diminishing positive parenting behaviors. These findings are consistent with research showing that severity of food insecurity/insufficiency is associated with a concomitant increase in levels of parental depression [37], [40]. However, this is the first study that we are aware of that has found medical need to be predictive of parental depressive affect and parenting behaviors. The finding of an association between food insufficiency and medical need predicting a reduction in positive parenting behaviors is in consonance with previous research that has found that material hardship has adverse effects on quality of parenting behaviors [8], [20], [41], [42]. This suggests that as dimensions and levels of material hardship increased, parents became less effective in their parenting practices and behaviors, while simultaneously attending to their children and trying to parent effectively.

Heightened levels of food insufficiency and medical need increases were associated with poor health status. These results support previous research showing that not having enough food to eat [26], [27], [31], and not having access to and utilizing medical care puts children at risk for poor health [35], [36], [45]. Together, these results suggest that material hardship has effects on parental factors and child health status; however, the effects of food insufficiency appear to be stronger than those of medical need. This provides some support for our contention that material hardship dimensions may have differential effects on parental factors and child health.

This study demonstrated that increased parental depression was associated with diminished positive parenting behaviors and poor health status; while positive parenting behaviors were associated with better child health. The finding of a correlation between parental depression and parenting behaviors is consonant with previous research that has found parental depression adversely affects the quality of parenting behaviors [3], [7], [9], [51], and has implications for child health [47], [48], [54]. The finding of an association between parental behaviors and child health status is in line with research that documented the effects of parental behaviors on child health [52], [55], [56]. Taken together, these findings suggest that parental depression has direct effects on child health, but some of those effects are explained through its association with positive parenting behaviors. Finally, in terms of the covariates, we found, consistent with previous research [58] that African American and Hispanic children were significantly more likely to be in poor health compared with Caucasian children.

### Limitations

Although the current study adds to the existing literature, there are some limitations to the study that may bear on our findings. First, we did not control for alternative sources of income, for example, participation in the food stamp program or receipt of assistance from informal sources. Receipt of formal or informal help may influence the degree of food insufficiency experienced. Second, we rely on parental reports to explore links among the constructs in the model. Thus, any potential parent-level bias is reflected in all the measures used in this study. The concern here is that parents who measure high on depression are more likely to report problems of every sort—food insufficiency, medical need, and poor parenting and children's poor health. Third, the NSAF items used to assess food insufficiency measured food insufficiency at the household level, and was not linked to an individual child. Thus, the extent of nutritional deprivation experienced by a child is hard to estimate. Fourth, it is important to recognize that our analyses focused on the effects of short-term income poverty, material hardships, and parenting factors on child health. As such the relationships observed may, therefore, be conservative in nature, and families experiencing prolonged poverty, and severe forms hardships need to be included in future studies addressing these relationships. Finally, the hypothesized associations among the constructs in this study are not the only relationships that could be used to examine the link between income poverty, material hardships and children's health. Alternative models specifying different relationships could be used to explore the links among income poverty, material hardships and children's health.

### Conclusions

With only cross-sectional data, we can conclude the following: first, family income matters for understanding child health. This is because we found that after adjusting for controls and other predictors, income poverty had an independent effect on child health status. Second, material hardship is a multi-dimensional construct, and is differentially associated with not only income poverty, but also with parenting factors and health status. Third, part of the effect of income poverty on child health is explained through its association with material hardship and parental factors. Fourth, part of the association between parental depression and child health status is explained through its relationship with parenting behaviors. And finally, income poverty, material hardship, and parenting factors are of consequence to child health to different degrees.

### Policy Implications

Our findings demonstrate that cash transfer programs aiming to increase families' financial resources are necessary because they may help improve children's physical well-being. However, our results show the complex interaction among the constructs in the model, and cash transfer alone may not result in parallel changes in the distribution of material hardship. This is borne out by the results showing that income explains a greater percentage of the variance in food insufficiency than medical need. It implies that income or cash transfers would be more beneficial for reducing food insecurity than for reducing medical need and increasing health care utilization. Thus, to reduce medical need and improve health care utilization, it would be necessary (in addition to cash transfer programs) to expand in-kind programs, such as, state or federal health insurance programs, because this is a more direct way to improve health care utilization. So, if the goal is to decrease medical need, using cash transfers alone may not result in improvement in health outcomes because such cash transfers could be appropriated for other purposes by families.

Because food insufficiency is detrimental to health, it means that in-kind programs such as the Food Stamp and other federal nutrition programs (e.g., School Breakfast and Lunch programs) for families and children ought to be continued, and if possible expanded so that the near-poor families could also benefit from those federal food programs. This may be another useful and direct approach to intervene and impact children's food and nutrient intakes to reduce the inimical effects of malnutrition on health.

In sum, our findings help to elucidate the multifaceted issues surrounding child health and points to the complexity that must be taken into consideration in both policy and practice. In particular, if the focus is on enhancing child health, then increasing family income and ameliorating material hardship, while concomitantly engaged in intervention efforts aimed at parental depression and parenting behaviors may have the greatest impact. In sum, our study suggests that more comprehensive approaches are needed to increase the likelihood of better health outcomes in childhood, rather than a single strategy that focuses solely on cash transfers or in-kind programs.
